# Determinants of continuing mental health service use among older persons diagnosed with depressive disorders in general hospitals: latent class analysis and GEE

**DOI:** 10.1186/s12913-022-08250-5

**Published:** 2022-07-11

**Authors:** Thida Mulalint, Acharaporn Seeherunwong, Napaporn Wanitkun, Sasima Tongsai

**Affiliations:** 1 D.N.S. Candidate, Faculty of Nursing, Mahidol University, Bangkok, Thailand; 2 Department of Mental Health and Psychiatric Nursing, Faculty of Nursing, Mahidol University, Bangkok, Thailand; 3 Department of Surgical Nursing, Faculty of Nursing, Mahidol University, Bangkok, Thailand; 4 Office for Research and Development, Faculty of Medicine Siriraj Hospital, Mahidol University, Bangkok, Thailand

**Keywords:** Attitude toward depression, Continuing mental health service use, Continuing service use, Depressive disorders, Drop out, Latent class analysis, Older persons, Mental health services delivery system, Marginal logistic regression model using generalized estimating equation (GEE), Service attention

## Abstract

**Background:**

Prevalence of depression in older persons was a leading cause of disability. This group has the lowest access to service and retention in care compared to other age groups. This study aimed to explore continuing mental health service use and examined the predictive power of the mental health service delivery system and individual factors on mental health service use among older persons diagnosed with depressive disorders.

**Methods:**

We employed an analytic cross-sectional study design of individual and organizational variables in 12 general hospitals selected using multi-stratified sampling. There were 3 clusters comprising community hospitals, advanced and standard hospitals, and university hospitals. Participants in each group were 150 persons selected by purposive sampling. We included older persons with a first or recurring diagnosis of a depressive disorder in the last 6 to 12 months of the data collection date. Data at the individual level included socio-demographic characteristics, Charlson Comorbidity Index, Attitude toward Depression and its treatment, and perceived social support. Data at the organizational level had hospital level, nurse competency, nurse-patient ratio, and appointment reminders. Descriptive statistics, Pearson chi-square test, latent class analysis (LCA), and marginal logistic regression model using generalized estimating equation (GEE) were used to analyze the data.

**Results:**

The continuing mental health service use among older persons diagnosed with depressive disorders was 54%. The latent class analysis of four variables in the mental health services delivery organization yielded distinct and interpretable findings in two groups: high and low resource organization. The marginal logistic multivariable regression model using GEE found that organizational group and attitude toward depression and its treatment were significantly associated with mental health service use (*p*-value = 0.046; *p*-value = 0.003).

**Conclusions:**

The findings suggest that improving continuing mental health services use in older persons diagnosed with depressive disorders should emphasize specialty resources of the mental health services delivery system and attitude toward depression and its treatment.

## Introduction

Depressive disorders (D.D.) in aging are not a usual way of life, but the prevalence rate has continually increased. This mental health problem can be solved by accessing services, medicine, and psychosocial therapy. Persons with D.D. who underwent treatment would have a better quality of life. The proportion of clients with high quality of life (QOL) increased from 28.5% to 42.5% after undergoing the treatment for one year [1]. Moreover, patient QOL scores rose in the 2nd month after antidepressant treatment and were stable in the 6th, 12th, and 24th, respectively [2]. However, previous studies reported that the rate of access to care in this age group was under compared to other age groups [3–5]. In addition, the rate of continuing to receive mental service use is essential to improve health outcomes but still is little understood.

For adults and older persons in six low- and middle-income countries, 69.5% of patients, have not received depression treatment in one year [6]. Across European regions in eight countries, 79% of late-life depression have received neither diagnosis nor treatment. Besides, a study in Korea found that the older persons were the lowest at receiving mental health services compared to other ages [7]. The evidence related to mental health service use in people with depression is most likely to explore access to health care services or adherence to antidepressants; on the contrary, continuing service use has received less attention. A study revealed that the dropout rate from mental health treatment was associated with higher age, mainly older persons. More than 50% of patients first diagnosed with depression drop out of service at the first time of appointment. Also, 25 and 15% did not return to receive mental health services at the second and third appointments [8].

Similarly, of 42% of Chinese patients with depressive disorders (mean age = 57.3, ≥ 55 years), 59% dropped out of depression treatment at the first month’s appointment, 71% dropped out by the second month, 77% at three months, 81% dropped out at six months, and 82% dropped out at 12 months [9]. We found no study about continuing or dropout rate of mental health service use in older persons with depressive disorders in Thailand. Continuing service use implies that the client continues to take antidepressants as treatment. The evidence has strongly supported that using antidepressant treatment as a protocol recommended would be remit effectively from depressive symptoms and improve QOL [10, 11].

Thailand’s Health policy has a strategic plan to increase the number of older people’s access to mental health services and recommends providing integrated physical and mental health services in general hospitals. A clinical practice guideline in public hospitals was provided, and trained physicians and psychiatric nurses to use the guideline 2015. The appointment for treatment of depression is at least six months after starting antidepressant drugs [12, 13]. As a result, depressive disorders’ access to services rose from 30 to 70% from 2015 to 2020 [14]. However, mental health service use in general hospitals has never been explored. A previous study revealed that older people have many barriers to health service utilization, both mental health service system and personal factors.

The evidence was inconclusive regarding distinguishing factors affecting mental health service usage among older persons with D.D. Various factors such as individual characteristics, social determinants, and clinical characteristics impact this service use outcome. For instance, females use mental health services more than males [15]. Having no spouse or significant others was likely to have lower treatment depression than those with marital status [16, 17]. Besides, comorbid conditions were negatively associated with treatment adherence [8, 16] and mental health service use with emotional problems [18]. Insufficient income [19], attitude toward depression, and embarrassment [20] were obstacles to receiving depression and negatively impacted service utilization.

In contrast, patients with a positive attitude toward depression and trust in nurses had more frequent health services [21]. Besides, social support plays an essential role in health care service utilization [22]. Moreover, the severity of depression was reported in both negative and positive associations with healthcare service utilization. For example, Grover, Dua, Chakrabarti, and Avasthi found that older persons with mild depression tended to drop out of the treatment at the early to intermediate stage of the treatment, and those with moderate depression were more likely to leave the medicine in the middle stage of treatment [23].

Meanwhile, Stein et al. found that older persons with more depressive symptoms (GDS ≥ 6) had 0.144 less medication treatment time (− 0.885, 0.378) compared to those with less severity of depression (GDS4–5) [24]. Hansen and Kristoffersen also found that severe depression of participants (30–87 years) was strongly associated with mental service use. Participants with severe depression utilized more mental services than those with moderate disease (OR 7.53, CI 2.75, 20.65) [25].

The mental health service delivery system is associated with mental health service use. It includes organizational and provider-related factors such as hospital level, nurse competency, nurse-patient ratio, and appointment reminders. These factors are the structure and organized delivery system for patients. However, far too little attention has been paid to mental health service organizations associated with mental health service use. Most studies have only been carried out in a small number of areas. For instance, Kales et al. indicated that primary hospitals predicted antidepressant treatment adherence of older persons with depressive disorders [16]. However, some studies have found relevance that can imply influencing factors at the hospital level and mental health services use. For instance, Germack, Bizhanova, and Roberts conducted a study with substantial hospital-level variation in remission rates among severe mental illness patients [26]. Another study based on patient perception also mentioned hospital levels and differences in the quality of services [27]. Similarly, providers’ performance is also helpful for improving care quality, and it was associated with mortality in a university hospital [28].

The conceptual framework in this study is derived from the Behavioral model of health service uses (B.M.), improving in the fifth phase [29]. The B.M. is a multilevel model that incorporates individual and contextual determinants of health services use. This model emphasizes that understanding health service use is best accomplished by focusing on contextual and individual determinants or characteristics. Unique characteristics consist of predisposing factors, enabling factors, and need. Personal characteristics can represent predisposing factors, including age, sex, and marital status. Enabling factors include sufficient income, social support, attitude toward depression, and its treatment. In addition, the need factor represents actual needs for health care services use. This study uses the diagnosis of depressive disorders as a classification according to ICD 10 and comorbidity in terms of index resource utilization [30].

Organization at the contextual level includes the amount and distribution of health services facilities and personnel and how they are structured to offer services [29]. In this study, organizational mental health service delivery comprises supplies of services in the hospital and the availability of service delivery that offers continuing services to older persons with D.D., including hospital-level, nurse competency, nurse-patient ratio, and appointment reminders.

Continuing mental health service use in this study means that older persons continuously attend mental health clinics by appointment and never miss a clinical appointment for depression treatment for more than 90 days. This meaning is consistent with the algorithm of D.D. management for Thai general practitioners that monitoring relapse should not leave attendance for more than three months [12]. Previous studies have little examined variance among the organizational and Individual determinants associated with continuing mental health service use. Therefore, this study aimed to (1) explore the rate of continuing mental health service use, (2) identify groups of organizations that took part in continuing mental health service use based on service delivery and human resources, and (3) explain organizational and personal determinants affecting continuing mental health service use within six months after diagnosed D.D. in general hospital among older persons.

## Methods

### Design

We conducted an analytic cross-sectional study with data on individual and organizational variables in 12 general hospitals in Thailand using multi-stratified sampling based on Thailand’s health service system administration [31]. There are 3 clusters comprised of community hospitals, advanced and standard hospitals, and university hospitals. To achieve an equal sample size in all groups, the number of participants in all clusters was 150. The hospitals in each grouping cluster were selected by purposive sampling.

### Sample size

Based on the rule of thumb concerning the relationship between sample size and model complexity, which also has some empirical support as the *N:q* rule, an ideal sample size-to-parameters ratio would be 20:1 [32]. This study has ten parameters, so the sample size is 10 × 20 = 200, and adjusting for design effect to compensate for the cluster sampling of 2; there were 200 × 2 = 400 cases [33]. Hence, the design effect’s final sample size was at least 400.

### Setting

Thailand is an upper-middle-income country located in South-East Asia. Policy and strategic plan for mental health service system in Thailand complied with WHO action plan and is making progress towards meeting the SDG 3. People at risk of D.D. are screened to provide early intervention. Older persons with chronic illness are identified as a high-risk group and are monitored by screening when they visit for annual service at the general hospital. Regarding the Thai health service system, available health facilities delivering services to people are classified according to a three-tier service system: primary, secondary, and tertiary care facilities [34]. The primary healthcare facility is the first contact individuals, families, and communities have with the health care system. Service provision emphasizes health promotion and prevention.

Secondary health care facilities provide curative care with initial referrals from primary care professionals. Health service provision is more complex and requires higher medical expertise. Both primary and secondary health facilities offer service at the community hospital under the authority of the Ministry of Public Health (MoPH). The tertiary health care facilities provide specialized investigation and treatment, usually on referral from primary or secondary medical care personnel. The facilities include MoPH general (standard-level) and regional (advance-level) hospitals. Mental health services for people with D.D. are usually provided treatment and care at secondary and tertiary care facilities because there are physicians to investigate, diagnose and prescribe antidepressant drugs. In addition, they have specialty mental health nurses to provide care to these groups of people. The university hospitals have service administration different than hospitals under the authority of MoPH. In this study, researchers classified hospitals into 3 clusters: community hospitals, advanced and standard hospitals, and university hospitals.

#### Continuing mental health service use and measurement

Continuing mental health service use in this study refers to continuing visits to the mental health clinic as the treatment plan or appointment attendance at mental health care service of older persons within six months after diagnosing the depressive disorders. Mental health service use was measured in two ways: the number of attendance times and continuity of mental health service uses. Firstly, the number of attendance times refers to the number of attendances by appointment at the mental health clinic of older persons within six months since the diagnosis date, excluding the first diagnosis date. Second, continuity of mental health services use refers to attending a mental healthcare appointment for older persons within six months after being diagnosed with depressive disorders. The respondence is categorized into non-continuing service use (score = 0) and continuing service use (score = 1). “Non-continuing service use” means that the older persons have a period of non-attending mental health service of 90 days or more since the diagnosis date. “Continuing service use” means that older persons attend mental health clinics continuously according to clinical appointments and never miss a clinical visit for depression treatment for more than 90 days.

#### Individual variables and measurement

Individual data were obtained for the Thai older persons aged 60 and over from the starting date of this study and the first diagnosis or recurrent of depressive disorders (F33) based on the 10th revision of the International Classification of Diseases (ICD-10) (F 32, F33, F34.1, F38, and F39) [35] since at least six months to one year before collecting data. The data were retrieved from electronic medical records of all hospitals providing care for depression to patients in Thailand. They had health insurance cards eligible for the same service and have lived in the community for at least six months. They were able to comprehend information in Thai, cooperate in the study, communicate by completing the Mini-cog Test on three or more occasions and be contacted by phone or address to make appointments for study participation. Participants were excluded from this study if they had been diagnosed with mania or bipolar conditions, had a severe somatic disease, could not answer questionnaires, or had suicidal thoughts.

Individual demographic and clinical variables were obtained from medical records, including age (60–69; 70–79; ≥80 years), sex (male; female), marital status (married; others), comorbidity, and severity of depression. Comorbidity was measured using the Charlson Comorbidity Index (CCI) [30], a measure of the incidence of 23 common diseases. The possible total scores ranged from 0 to 42. The higher the score, the more severe burden of comorbidity and the greater the average yearly cost per patient. The severity of depression was classified into four diagnostic groups based on the criteria of the ICD-10 [35]: mild depressive symptoms (F32.0 and F33.0); moderate depressive symptoms (F32.1 and F34.1); severe depressive symptoms (F32.2 and F32.3), and unspecified or depression not otherwise specified (NOS) (F32.9, F38, and F39). Perceived sufficient income was assessed by asking about the perceived adequacy of the money earned from a person, company, or government over a period for daily living or a particular purpose (sufficient income; insufficient income). Attitude toward depression and its treatment (ATDS) and social support were assessed by interviewing older persons diagnosed with depressive disorders via standard questionnaires. ATDS was measured by attitude toward depression and a depression treatment scale [36]. The original ATDS consisted of a 27-item questionnaire with a Likert scale of 1 (strongly disagree) to 5 (strongly agree). The researchers translated the ATDS into Thai (ATDS-T) following WHO’s tool translation instrument [37]. The ATDS-T utilized 25 items from the original 27-item ATDS scale with qualified content validity and reliability. The reliability of the ATDS-T was .70 (*n* = 30). Perceived social support was measured using the Multidimensional Scale of Perceived Social Support (MSPSS) [38] and translated into Thai by Wongpakaran Tinakon and Wongpakaran Nahathai [39]. The MSPSS-Thai version consists of 12-items with a Likert scale of 1 (very strongly disagree) to 7 (very strongly agree) and overall positive opinions. The measurement consists of 3 sources of support, including family support, friend support, and significant-other support.

#### Organizational variables and measurement

Organizational characteristics deliver the mental health services relevant to patient care for depression. Administrative information of the 12 hospitals was obtained by interviewing a nurse of a mental health clinic or mental health outpatient clinic that had experienced care provision at an outpatient unit for at least five years or had experience caring for psychiatric patients for at least two years. The researchers developed an interview questionnaire for collecting information about organizational characteristics. The questionnaire was content validated by three experts in mental health services. Organizational attributes in this study consisted of hospital-level, nurse competency, nurse-patient ratio, and use of appointment reminders. **Hospital-level** was categorized according to mental health care resources in Thailand (university hospitals, advanced and standard hospitals, and community hospitals) [34, 40]. A university hospital refers to a health care facility where the institute is under the university’s authority. This health care facility combines health services with medical students’ education and medical research. A university hospital provides health services to patients with highly specialized staff, technical equipment, and complete mental health teams. Advance-level hospitals refer to regional hospitals with at least 500 beds and a comprehensive set of specialists on staff. These hospitals are capable of tertiary care.

The standard-level hospitals refer to general hospitals with 200 to 500 beds to support patients requiring specialized, complex treatments. The hospitals are located in provincial capitals or central districts and are capable of secondary care. The advanced and standard-level hospital is a high-referral-level hospital with highly specialized staff and technical equipment. Besides providing general health services, the hospital offers mental health services to patients with acute psychiatric problems through multidisciplinary psychiatric teams, outpatient psychiatry clinics, or inpatient departments. Community hospitals are located at the district level and are usually limited to primary care treatment. These will refer patients needing more advanced or specialized care to general or regional hospitals. There is often only one general practice physician at this hospital. **Nurse competency** refers to the complex integration of knowledge, including professional judgment, skills, values, and attitudes based on the holism perspective [41]. This study’s integrated nursing competency is ensured through formal nursing graduation and training in nursing specialty programs. These programs have curriculum goals focused on developing nursing competency at three different levels of education and training available in Thailand. The first group consists of 1) registered nurses (RN), 2) RN+ with a specialty in psychiatric and mental health nursing (PMHN), and a specialty in gerontological nursing (GERO-N). The second group consists of those with 1) R.N., 2) R.N. + PMHN/GERO-N, and 3) R.N. + master’s degree of nursing science (MNS). The last group consists of 1) R.N., 2) RN + PMHN/GERO-N, 3) RN + MNS, and 4) RN+ an advanced practitioner nurse (APN). **The nurse-patient ratio** refers to the number of patients with mental illness/problems who received mental services in the outpatient department of the hospitals per day divided by the total number of nursing staff on duty providing care to those patients on that day. **Appointment reminders** are defined as strategies of reminders on the date of appointment attendance in mental health clinics (non-reminder; reminder before the appointment date by phone or text message).

### Data collection

The data collection procedure included: firstly, extracting data of older persons with depressive disorders: the researchers, assistant researchers, and medical technology officers retrieved patient data from the hospital database (medical history, address, and phone number). Second, the researchers reviewed the medical records to gather several patients who met the inclusion criteria. Thirdly, 1584 eligible patients were identified. Subsequently, co-researchers enlisted contactable patients to participate with 526 cases or 33.2% (526 of 1584) of eligible patients. Seventy-five contactable patients, or 14.3% (75 of 526), could not participate due to illness or their caregiver was not comfortable providing information. Finally, 85.7% (451 of 526) were willing to participate in this study. Various data collection methods were considered for maximum data acquisition based on the availability and convenience of participants and the health care settings. Data collection included 188 cases or 41.7% with a telephone interview, 152 cases or 33.7% with home visits, 47 patients or 10.4% responses by mail, and 64 cases or 14.2% face-to-face interviews at the hospital. Data completeness was verified after finishing the process of collection. Because 17 cases or 3.8% (17 of 451) had been diagnosed within two years, and 12 cases or 2.7% (12 of 451) were referred to health care services near their homes, 29 patients or 6.0% (29 of 451) were eliminated. Thus, finally, participants were 424 cases, or 94.0% (424 of 451). Nevertheless, the number of participants had remained representative of the population studied, as seen in Table 1.

**Table 1:** Demographic characteristics of the participants categorized by hospital level (*n* = 424)

Characteristics	Hospital level						Total	
	University hospital		Standard and advanced hospital		Community hospital			
	Count	%	Count	%	Count	%	Count	%
Participants	137	32.3	147	34.7	140	33.0	424	100.0
Age group								
60–69 years	89	65.0	83	56.5	80	57.2	252	59.4
70–79 years	37	27.0	48	32.6	44	31.4	129	30.4
≥ 80 years	11	8.0	16	10.9	16	11.4	43	10.2
Sex								
Male	35	25.6	47	32.0	51	36.4	133	31.4
Female	102	74.4	100	68.0	89	63.6	291	68.6
Marital status								
Single	20	14.6	4	2.7	4	2.9	28	6.6
Married	64	46.7	81	55.1	79	56.4	224	52.8
Widowed	33	24.1	49	33.3	47	33.6	129	30.4
Divorced	19	13.9	13	8.9	7	5.0	39	9.2
Priest	1	.7	0	0	3	2.1	4	1.0
Medical service payment								
No use	26	19.0	0	0	1	.7	27	6.4
Government reimbursements	66	48.2	49	33.3	14	8.9	129	30.4
Social Security	5	3.6	2	1.4	1	.7	8	1.9
Universal Coverage (UC)	40	29.2	96	65.3	125	86.7	260	61.3
Sufficiency income								
Insufficiency	48	35.0	71	48.3	89	63.6	208	49.1
Sufficiency	89	65.0	76	51.7	51	36.4	216	50.9

### Ethics

This study was reviewed and approved by the institutional review board of the Research Ethics Committee of the Faculty of Medicine, Siriraj hospital (COA No. Si527/2019), Medicine Ramathibodi Hospital, Mahidol University (COA No. MURA2019/1264), and the Committees of all selected hospitals under the Ministry of Public Health. Participants were provided information explaining the study before providing written informed consent. All study procedures followed ethical guidelines and regulations. The data collection period started in May 2018 and went to November 2020.

### Statistical analysis

Data management methods validated data entry by analyzing missing data, outliners, frequencies, means, standard deviations, and ranges of scores on each variable. Descriptive statistics were used to analyze categorical and continuous data. Categorical data included sex, marital status, sufficient income, the severity of depression, mental health service delivery system (hospital level, nurse competency, nurse-patient ratio, appointment reminder), mental health service use, and the continuity of care reported as frequency and percentage. Continuous data such as age, comorbidity, attitude toward depression, and social support were reported as mean and standard deviation (S.D.) or median, and range as appropriate. Latent class analysis (LCA) identified mental health service delivery systems based on contextual factors (hospital level, nurse competency, nurse-patient ratio, and appointment reminder). Four indices were used to select the correct number of latent classes, i.e., Bayesian information criterion (BIC), Akaike information criterion (AIC), Pearson’s chi-square goodness of fit (χ^*2*^), and Likelihood ratio/deviance statistic (*G*^*2*^). Lower BIC, AIC, χ^*2,*^ and *G*^*2*^ values indicate a better model fit. However, BIC for LCA models is a good indicator for class enumeration over the rest [42]. Description for each group was agreed upon through discussion with the research team. A marginal logistic regression model using a generalized estimating equation (GEE) for within-subject correlation between patient outcomes within the same hospital was performed for associating groups of mental health service delivery systems and individual characteristics with mental health service use.

## Results

Of the total 424 participants, of which 32.3% (*n* = 137) were from the two university hospitals, 34.7% (*n* = 147) were from five advanced and standard hospitals, and 33% (*n* = 140) were from five community hospitals across four regions in Thailand. The demographic and clinical characteristics of the participants are shown in Tables 1 and 2.

**Table 2:** Clinical characteristics of the participants categorized by hospital level (*n* = 424)

Comorbidity	Hospital level						Total (***n*** = 424)	
	University hospital (***n*** = 137)		Advanced and standard hospital (***n*** = 147)		Community hospital (***n*** = 140)			
	Count	%	Count	%	Count	%	Count	%
**Comorbidity**								
No comorbidity	56	40.9	63	42.9	59	42.1	178	42.0
≥ 1disease	81	59.1	84	57.1	81	57.9	246	58.0
**No. of disease**								
1 disease	32	23.4	44	29.9	37	26.4	113	26.7
2 diseases	27	19.7	26	17.7	29	20.7	82	19.3
3 diseases	17	12.4	11	7.5	10	7.1	38	9.0
≥ 4 diseases	5	3.6	3	2.0	5	3.5	13	3.3
**Top 10 Comorbidity**								
No disease	56	40.9	63	42.9	59	42.1	178	42.0
Hypertension	64	46.7	63	42.9	57	40.7	184	43.4
Diabetes	24	17.5	20	13.6	27	19.3	71	16.7
Ulcer disease	2	1.5	11	7.5	13	9.3	26	6.1
Cerebrovascular disease	8	5.8	10	6.8	7	5.0	25	5.9
Moderate/ severe renal disease	7	5.1	6	4.1	12	8.6	25	5.9
Chronic pulmonary disease	9	6.6	5	3.4	7	5.0	21	5.0
Any tumor	17	12.4	2	1.4	1	0.7	20	4.7
Mild liver disease	5	3.6	4	2.7	2	1.4	11	2.6
Congestive heart failure	0	0.0	7	4.8	3	2.1	10	2.4
Take warfarin /coumadin	5	3.6	2	1.4	3	2.1	10	2.4
**Severity of depressive symptoms**								
Mild depression (F32.0, F33.0)	5	3.6	8	5.4	76	54.3	89	21.0
Moderate depression (F32.1, F34.1)	40	29.2	31	21.1	29	20.7	100	23.6
Severity depression (F32.2, F32.3)	48	35.0	93	63.3	6	4.3	147	34.7
NOS/F32.9, F38, F39	44	32.1	15	10.2	29	20.7	88	20.8

### Continuation of mental health service use

According to Table 3, more than half of the participants were in continuous mental health service. University hospitals’ mental health service use had the most continuous mental health service (39.3%). Of the participants who did not have ongoing mental health services, 37.9% were participants whose mental health services use was in advanced and standard hospitals, the same amount as in community hospitals.

**Table 3:** The hospital-level and participant characteristics categorized by mental heal service use

	Mental health service use				Total (424)	
	Not continue (***n*** = 195; 46%)		Continue (***n*** = 229; 54%)			
	Count	%	Count	%	Count	%
Hospital level						
University hospital	47	24.2	90	39.3	137	32.3
Advanced and standard hospital	74	37.9	73	31.9	147	34.7
Community hospital	74	37.9	66	28.8	140	33.0
Sex						
Male	60	30.8	73	31.9	133	31.4
Female	135	69.2	156	68.1	291	68.6
Age groups						
60–69 years	115	59.0	137	59.8	252	59.4
70–79 years	59	30.3	70	30.6	129	30.4
≥ 80 years	21	10.8	22	9.6	43	10.1
Marital status						
Other	89	45.6	111	48.5	200	47.2
Married	106	54.4	118	51.5	224	52.8
Perceived income						
Insufficient	106	54.4	102	44.5	208	49.1
Sufficiency	89	45.6	127	55.5	216	50.9
Severity of depression						
Mild depression	46	23.6	43	18.8	89	21.0
Moderate depression	39	20.0	61	26.6	100	23.6
Severe depression	68	34.9	79	34.5	147	34.7
F32.9/NOS	42	21.5	46	20.1	88	20.7

Regarding the number of appointment attendance times within six months, Table 4 shows that only a few participants had completed attendance eight times (0.7%), and most participants only attended two times (26.4%). In addition, 13.9% of participants did not even show up for their first appointment.

**Table 4:** Number of times to attend by appointment within six months categorized by hospital level

Number of visits	Hospital level						Total (***n*** = 424)	
	University hospital (***n*** = 137)		Advanced and standard hospital (***n*** = 147)		Community hospital (***n*** = 140)			
	Count	%	Count	%	Count	%	Count	%
0	14	10.2	26	17.7	19	13.6	59	13.9
1 time	16	11.7	31	21.1	31	22.1	78	18.4
2 times	37	27.0	36	24.5	39	27.9	112	26.4
3 times	35	25.5	22	15.0	30	21.4	87	20.5
4 times	21	15.3	17	11.6	16	11.4	54	12.7
5 times	7	5.1	7	4.8	4	2.9	18	4.2
6 times	3	2.2	5	3.4	0	0.0	8	1.9
7 times	3	2.2	2	1.4	0	0.0	5	1.2
8 times	1	0.7	1	0.7	1	0.7	3	0.7

### Groups of mental health service delivery system

The latent class analysis analyzed each group of organizations based on the mental health service delivery system (hospital level, nurse competency, nurse-patient ratio, and appointment reminder). Table 5 shows the model fit statistics of latent class analysis for deciding the number of classes. Comparing the results obtained from the latent class analysis for the model with 2, 3, and 4 latent variables, we find that when using information criteria (AIC and BIC), the best model is the one with 3 or 4 latent classes (AIC = 2446.32, BIC = 2964.69 and AIC = 2312.34, BIC = 3004.84, respectively), as the minimal value of those criteria indicate the best fit of the model [42]. The lower BIC and AIC values indicate a better model fit. However, the two-class solution with the highest BIC was slightly larger than the lowest BIC in the three-class solution. It was chosen as the optimal solution as it yielded classifications that were distinct and interpretable and had adequate class sizes. The two latent classes were the best in the current study for a clear interpretation of the mental health service delivery system.

**Table 5:** Model- fit statistics of latent class analysis models for number of classes

Number of classes	AIC	BIC*	G^**2**^	χ^**2**^
2	2917.67	3261.90	820.08	1239.85
3	2446.32	**2964.69**	262.74	296.12
4	**2312.34**	3004.84	**42.75**	**29.83**

Bold = Ideal class model based on model-fit statistics; Shaded = Class model selected based on interpretation of organization of mental health service delivery system class

*AIC* Akaike information criterion, *BIC* Bayesian information criterion; χ^*2*^ *=* Pearson’s chi-square goodness of fit; *G*^*2*^ = Likelihood ratio / deviance statistic

*BIC for LCA models is a good indicator for class enumeration over the rest^1^

### Characteristics of groups of the mental health service delivery system

According to latent class analysis, Table 6 shows that the organization of the mental health service delivery system was divided into two classes. Class one was a high potency mental health services system resource. It included two university-level hospitals, with 77.4% of the participant in Siriraj and Ramathibodi Hospitals, and one standard and advanced hospital (26.6%), Chaophraya Yommarat Hospital. The nurses with the highest qualification in mental health clinics were 100% R.N.s combined with R.N.s + MHN/MNS. This class had a high workload, between 23.92 and 37.3, with a nurse-patient ratio of 37.5 for 40.1%, a nurse-patient ratio of 23.92 for 37.3%, and a nurse-patient ratio of 16.38 for 22.6%. In addition, the high resource system had the most successful appointment reminder system for older persons who would visit mental health services. It had due date reminders of 77.4%, with only 22.6% without reminders. The characteristics of organization group two were low potency of mental health service resources. It was 43.3% standard and advanced hospitals and 56.7% of community hospitals. However, the low resource group had higher qualifications than the high resource group. Of nurses, 61.1% consisted of RN + (RN+ PMHN), RN+ (RN + MNS/APN). Moreover, 38.9% of registered nurses had been trained to provide psychiatry nursing care.

**Table 6:** Organizational characteristics categorized by the group of organizations (*n* = 424)

Characteristic	Group of Organization			
	High resource (***n*** = 177)		Low resource (***n*** = 247)	
	Count	%	Count	%
**Hospital level**				
University hospital	137	77.4		
Advanced and standard hospital	40	22.6	107	43.3
Community hospital			140	56.7
**Hospital name**				
Chaoprayamaraj hospital	40	22.6		
Khonkean hospital			30	12.2
Kuangnai hospital			44	17.8
Lamphun hospital			25	10.1
Mousamsip hospital			16	6.5
Pathaluang hospital			24	9.7
Rama hospital	71	40.1		
Sappasitiprasong hospital			28	11.3
Siriraj hospital	66	37.3		
Srimuangmai hospital			14	5.7
Tragarn hospital			22	8.9
Warinchamrap hospital			44	17.8
**Nurse-patient ratio**				
9.78			25	10.1
10.75			44	17.8
11.00			30	12.1
11.40			28	11.3
14.75			44	17.8
16.38	40	22.6		
17.33			24	9.7
20.00			16	6.5
21.33			22	8.9
23.92	66	37.3		
36.25			14	5.7
37.50	71	40.1		
**Nurse competency**				
RN + PMHN			96	38.9
RN + PMHN+MNS	177	100.0		
RN + PMHN+MNS/APN			151	61.1
**Appointment reminders**				
No	40	22.6	247	100.0
Yes	137	77.4		

The low resource class had a high workload or lower nurse-patient ratio than the high resource class. Thereby nurse-patient ratio 10.75 was 17.8%, ratio14.75 was 17.8%, ratio11.0 was 12.1% and ratio14.75 was 17.8%. In addition, the low-resource mental health service group does not have any reminder system to alert patients to mental health care services.

Table 7 shows that the older persons with depressive disorders in the high resource organization were statistically significant more continuous mental health service use (*n* = 112, 63.3%) than low resource organization (*n* = 117, 47.4%) with a *p*-value of 0.001.

**Table 7:** Continuous mental health service use of participants within six months categorized by group of organization (*n* = 424)

	Group of organization				***p***-value
	High resource (***n*** = 177)		Low resource (***n*** = 247)		
	Count	%	Count	%	
**Mental health service use**					0.001
Not-continuing	65	36.7	130	52.6	
Continuing	112	63.3	117	47.4	

### Organizational and individual determinants affecting continuing mental health service use

According to Table 8, logistic models run sequentially to assess mental health service use prediction over six months. Within six months, individual characteristics were significantly associated with mental health service use by the organizational group. When an organized group was added to the univariate model, the organized group was significantly related to mental health services. The high resource organization had 1.9 times increase in odds of mental health service use (unadjusted Log OR 0.637, *p*-value = 0.007; 95% CI: 0.171, 1.103) compared with the low resource organization. In the multivariable model, the high resource organization remained significantly associated with mental health service use after adjustment for other individual characteristics (adjusted Log OR 0.511, *p*-value = 0.046; 95% CI: 0.009, 1.014) versus the low resource organization.

**Table 8:** Factors associated with mental health service use within six months in older persons with depressive disorders (*n* = 412)

Factors	Univariable			*p*-value	Multivariable			*p*-value
	Unadjusted Log OR	SE	95% CI		Adjusted Log OR	SE	95% CI	
Organization group								
Low resource	Reference				Reference			
High resource	0.637	0.238	(0.171, 1.103)	0.007	0.511	0.256	(0.009, 1.014)	0.046
Age (years)	−0.12	0.013	(−0.038, 0.013)	0.333				
Sex								
Female	Reference							
Male	0.098	0.149	(−0.194, 0.391)	0.510				
Marital status								
Other	Reference							
Married	−0.056	0.188	(−0.425, 0.313)	0.765				
Perceived income								
Insufficient	Reference				Reference			
Sufficient	0.337	0.143	(0.057, 0.617)	0.018	0.310	0.161	(−0.005, 0.626)	0.054
ATDS	0.021	0.007	(0.007, 0.035)	0.003	0.021	0.007	(0.007, 0.035)	0.003
MSPSS	0.008	0.006	(−0.004, 0.020)	0.184				
CCI	0.025	0.036	(−0.045, 0.096)	0.482				
Severity of depression								
Mild	Reference							
Moderate	0.299	0.326	(−0.340, 0.938)	0.359				
Severe	0.108	0.270	(− 0.421, 0.638)	0.688				
F32.9/NOS	−0.299	0.283	(−0.854, 0.256)	0.291				

*OR* Odds ratio, *CCI* Charlson Comorbidity Index, *ATDS* Attitude toward depression and its treatment scale, *MSPSS* Multidimensional Scale of Perceived Social Support. Adjusted model control for organization group and individual characteristics that might affect the response, including sex, marital status, perceived income, and severity of depression

Regarding individual characteristics, perceived sufficiency income was only associated with mental health service use in the univariate model (unadjusted Log OR 0.337; 95% CI: 0.057, 0.617) versus insufficient income. ATDS was significantly associated with mental health service use within six months. In the univariate (unadjusted Log OR 0.021; 95%CI: 0.007, 0.035) and multivariable model, after adjustment for other individual characteristics and organizational groups, ATDS remained significantly associated with mental health service use within six months (adjusted Log OR 0.021, *p*-value = 0.003; 95%CI: 0.007, 0.035).

## Discussion

The present study is the first study to clarify the predictive power of the mental health service delivery system and individual determinants on continuing mental health services use among older persons with D.D. Regarding mental health service use of the older persons with D.D. in the last six months after diagnosis of D.D., the dropout rate at the first appointment was 13.9%. Dropout was less than in a previous study of older Indian persons with D.D. when 41.4% (58/140 persons) never returned after first attending psychiatry outpatient facilities of a tertiary hospital [23]. The current study had a range of dropouts depending on the type of hospital facility., The dropout rate ranged from 10.2 to 17.7%, with the lowest rate in university hospitals and the highest rate in advanced and standard hospitals comparable to tertiary hospitals in India. The advanced and standard hospitals are the referral hospitals from the community hospital. The possible reason for appointment non-attendance may be transportation and time constraints.

In contrast, the causes for early dropout of Indian older persons were “no relief” of symptoms, closely followed by complete relief of symptoms [23]. In addition, the rate of not attending mental health clinics at least 90 days consecutively out of six months after diagnosis in this study was 46%. The highest rate was in the advance and stardard hospital and community hospital (37.9%). The dropout rate among Indian older persons was 23.6% after three months and 8.6% between three and six months. These findings show different appointment attending behaviors in older persons of two cultures, with Indian older persons likely to drop out in earlier scheduled appointments and Thai older persons to drop out later. The reasons for non-attendance in the current study were attitudes toward depression and its treatment, insufficient income, and factors related to organizational resources for delivery services.

The characteristic of organizations affected continuing mental health service use. Older persons were more likely to continue appointment attendance at the high resource organizations (63.3%) than the low resource organizations (47.4%). It is possible that high-resource organizations (with highly qualified nurses, multidisciplinary professionals, more choices of antidepressant drugs, and psychosocial therapies) can provide services that better meet the need of mental health service users. Previous studies have reported that mental health service users prioritize access, information, peer support, service avoidance, and day centers as continuing care elements [43]. Furthermore, service users avoided service use because they did not realize they needed support or feared the loss of choice and control. Some avoidance service users had developed their strategies for living and no longer wanted or needed services [43].

After adjusting for all other determinants, the determinants affecting continuing mental health service use among older persons with D.D. were high resource organizations and ATDS. The two determinants are modifiable factors associated with depression treatment adherence among older persons [44]. The finding reinforces a prior study [45] that the availability of specialized mental health services such as combined therapy and psycho-pharmacotherapy assists in retaining depression treatment among ethnic and racial minorities in the United States. Also, it is consistent with findings of a previous study that medical facilities were associated with a four-month adherence among older persons with D.D [16]. Furthermore, older persons who received a prescription in primary care were significantly more likely to be non-attendance than those who received service in psychiatric hospitals [16]. According to ATDS, in this study, the ATDS in the older persons tended to be high, including acceptance of treatment, perceived stigma and shame, negative to antidepressants, self-stigma, and preferred psychotherapy. In this study, the ATDS in older Thai persons was consistent with prior research revealing that older Americans had positive help-seeking attitudes or positive beliefs about the efficacy of treatment for mental health problems [46]. The older persons with a better attitude toward depression and its cure had a significantly higher mental health service use than those with less ATDS. The result supports Kohls et al. [47] finding that people in countries with depression awareness campaigns on personal stigma, perceived stigma, openness to help, and perceived value of help reported more willingness to seek professional help than respondents unaware of it. This study demonstrated that the mechanism of organization groups was a variable influence on the individual characteristics. This principle underpins the Behavioral Model of health service uses (B.M.), improving in the fifth phase [29] that applies organization groups as the contextual characteristics. This study emphasized only organization groups and ATDS promoting mental health services use. Therefore, as discussed above, this study demonstrated the mechanism and evidence of the Behavior model of health services for investigating factors predicting mental health service use among older persons with depressive disorders. This finding contributes to discussing modifying resources of organization and ATDS among policymakers to improve appointment attendance among older persons with D.D.

## Limitations

This naturalistic study obtained data from medical histories of hospital databases, followed by interviews with older persons, so it has some restrictions. We acknowledge several limitations of our investigation. First, the operational definition of mental health service use is interpreted in the form of dichotomous categories. It may be more accurate for the researchers to calculate the non-appointment attendance ratio per appointment schedule over six months. Also, some physicians may not follow appointment adherence in the depression management protocol. Second, there was no information from patients on their reasons for non-appointment attendance in this study. Further study should ask for the reasons for non-attendance or for keeping the attendance plan. Third, this study’s group of organizational determinants comes from the LCA of four variables. Other unknown determinants such as provider-patient communication, type of depression treatment, provider productivity, etc., may have influenced continuing mental health service use. The LCA identifies subgroups or classes of characteristics of the mental health service delivery system on the balance of similarities and differences among some of the variables in the study [48]. Therefore, assigning different variables may give answers to a different number of groups or group characteristics. However, the two classes of the mental health service delivery system identified by LCA in the present study can distincguish the continuing mental health service use and giving a clear interpretation of the mental health service delivery system. Fourth, because of no available score or level of severity of depressive symptoms in the medical records, we used the depression diagnosis as classified by ICD-10 to represent need factors of the B.M. model. The classification of depression diagnosis may be biased as to the actual severity of depressive symptoms [49]. Fourth, our population was restricted to older persons whose depression was first diagnosed or was a recurrent depression within one year and had information on appointment attendance in the same hospital. Therefore, the patients referred for care from another hospital remained beyond our scope.

## Conclusion

The continuing mental health service use among older persons diagnosed with depressive disorders was 54%. This study provides evidence regarding organizational and individual level determinants of continuing mental health service use in various facilities of general hospitals in Thailand. LCA identified two variables based on hospital level, nurse-patient ratio, nursing competency, and appointment reminders, namely “high and low resource organizations.” After adjusting for determinants and group of organization, while controlling for within-hospital correlation using GEE, the finding highlight high resource organization as the health service delivery system determinant and the ATDS as the individual determinant that contributes most to continuing mental health service use among older persons with D.D.

## Data Availability

The datasets used and analyzed during the current study are not publicly available due to the used data protection declaration. Requests for reasonable use should be sent to the Dean of Faculty of Nursing, Mahidol University.

## Abbreviations

APN: Advanced practitioner nurse
ATDS: Attitude toward depression and its treatment
B.M.: Behavioral model of health service uses
D.D.: Depressive disorders
GEE: Marginal logistic regression model using generalized estimating equation
GERO-N: Specialty in gerontological nursing
LCA: Latent Class Analysis
MNS: Master’s degree of Nursing Science
MSPSS: Multidimensional Scale of Perceived Social Support
PMHN: Specialty in psychiatric and mental health nursing
QOL: Quality of life
R.N.: Registered nurses
